# Challenges for the European governance of synthetic biology for human health

**DOI:** 10.1186/s40504-014-0006-7

**Published:** 2014-03-20

**Authors:** Conor MW Douglas, Dirk Stemerding

**Affiliations:** Technology Assessment, Rathenau Institute, The Hague, 2593 HW,, The Netherlands; Collaborations for Outcomes Research and Evaluation, Faculty of Pharmaceutical Sciences, University of British Columbia, 2405 Wesbrook Mall, Vancouver BC V6T 1Z3,, Canada

**Keywords:** Synthetic biology, ELSI, Governmental regulation, Anticipatory governance, Responsible research and innovation

## Abstract

Synthetic biology is a series of scientific and technological practices involved in the application of engineering principles to the design and production of predictable and robust biological systems. While policy discussions abound in this area, emerging technologies like synthetic biology present considerable challenges in the articulation of concrete policy options given that their introduction into society may still be in the distant future. This paper reports on a series of governance workshops that focused on synthetic biology’s ethical, legal, and social implications (ELSI) as they pertain to human health, and discusses particular limitations of the ELSI approach that we encountered in our work. In an attempt to avoid policymaking for potential implications of uncertain future applications we instead conclude by proposing tangible forms of anticipatory governance that may be more adequate in addressing the more immediate concerns raised by synthetic biology.

## Introduction: Synthetic biology, and its ethical, legal and social implications

In this article we want to share our experiences with policy-oriented work that was undertaken in the context of EU-funded research that has come to typify ELSI approaches to the life-sciences. These experiences were gained over the course of a three-year European (FP7 Science in Society) project *Synthetic Biology for Human Health – The Ethical and Legal Issues* (SYBHEL). It was our original task in this project to add to the existing policy literature on synthetic biology (SynBio) by exploring the *governance implications* of the ethical, legal, and social issues arising from potential future applications of SynBio in the field of human health. However, in trying to complete this task we faced a number of challenges that made us reflect on the aims of current forms of ELSI scholarship on new and emerging technologies like SynBio. As we will show in this article, the main findings of our work in the SYBHEL project also prompted us to reconsider the traditional aims of doing ELSI research.

SynBio is an emerging field that combines molecular biology, engineering, biochemistry, computer sciences, and other disciplines into new practices of biological design that are both diverse in their approaches and full of hope and promise. While SynBio can be seen as a collection of activities rather than a distinct, singular, or totally novel practice, some of its proponents claim that such activities stand to revolutionize our mode of industrial production through the assemblage of multipurpose micro-organisms, based on sub-cellular standardized biochemical parts that may or may not exist in nature (Carlson 2007; Singh 2011). In debates about the novelty of SynBio, some define it as an extension of earlier practices in genetic engineering. Others argue that those earlier practices could rather be seen as genetic modification; whereas current activities really and truly aim to apply *engineering principles* of predictability, reliability, control, and abstraction to the construction of various kinds of biological systems (Tucker and Zilinskas 2006; O’Malley et al. 2008). However, these visions generally agree on the expectation that SynBio, in adopting engineering principles, may foster new options for bio-based innovation in materials, energy, food, environmental remediation, and medicine (Lowerie and Tait 2010; Cichocka *et al*. 2011).

These visions of SynBio have also given rise to a wider debate about its societal implications. Over the past ten years this debate has led to a flurry of activity – particularly in Europe – on science policy analysis in the form of reports and opinions by ethical committees, learned societies, as well as governmental and nongovernmental organizations (Stemerding and Rerimassie 2013). To date these policy discussions have tended to focus on SynBio in general and to a series of ethical, legal and social issues that have come to characterize a kind of ‘ELSI scholarship’ on new and emerging technologies (Calvert and Martin 2009; Rabinow and Bennet 2009 and Rabinow and Bennett 2012). Typically these reports and opinions start with a definition of SynBio and its related techniques and technologies, and then provide a series of potential applications that are envisaged across a number of different sectors, which are subsequently contrasted against concerns about possible risks and implications. Predominantly these issues take the form of biosafety and biosecurity risks relating to the accidental and purposeful release of SynBio products, the role of intellectual property regimes and patenting in securing or hindering a fair development of SynBio, and wider issues of ethical and public acceptability raised by the engineering ambitions of SynBio.

As a research activity which focused on the *ethical and legal implications* of potential future applications of SynBio for human health, SYBHEL was designed and funded as a typical ELSI project. However, over the course of our work we found that this ELSI approach was not particularly amenable to the formulation of actionable policy recommendations to the European Commission. This was in part due to the future-orientated nature of SynBio’s applications, which are highly uncertain. As a result, our policy-orientated ELSI research was being founded on scientific speculations about the great potential of future technologies, and on this basis engaging in ‘speculative ethics’ (Nordmann 2007). Moreover, by focusing on the ‘downstream’ implications of potential future developments that are envisaged for SynBio we found that we were taking for granted current practices and developments actually on-going in the early phases of the emerging fields. Thus, working in a typical ‘ELSI-mode’, we were confined to following the science rather than engaging ourselves in issues of science and technology policy making.

In the following we will show how our experiences in the SYBHEL project involved us in an important challenge: bridging the gap between innovation and ELSI by paying attention to ongoing developments in research and innovation (Rip and van Lente 2013). In this way, our work also demonstrates a development noted by Chadwick and Zwart in their introductory editorial to *Life Sciences, Society and Policy,* involving a shift from ELSI – as research focusing on ethical, legal and social implications – to a form of *responsible research and innovation* that calls ‘upon research consortia to make these reflections and deliberations an integrated part of their work’ (Chadwick and Zwart 2013: 2–3). We conclude the paper by proposing tangible forms of anticipatory governance that may be more adequate in addressing the more immediate concerns raised by SynBio.

## Synthetic biology and human health – ethical and legal issues: The SYBHEL project

Our aim to address the policy implications of the ethical, legal, and social issues that might arise from future SynBio health applications was defined by a specific policy work-package within the SYBHEL project. Our task was to synthesize the findings of the other SYBHEL work-packages that explored the ethical and legal issues pertaining to SynBio’s health applications, and place them in a policy-relevant context. In approaching this task we asked descriptive questions about the kind of SynBio *health applications* that are envisioned in the field, the possible *ethical and legal issues* related to those applications, and the *governance tools* available for addressing these questions in the context of European health policy making. In doing so, our approach was inspired by the above-mentioned reports and opinions on SynBio more generally, and can broadly be seen as following the ELSI mode that has come to characterize much of the policy work on new and emerging technologies.

In order to explore and understand the manner by which SynBio and governance could interact in a meaningful way to achieve (public) health goals, we organized two expert workshops, one focusing on the European context, the other on a global health context (Douglas and Stemerding 2011, Douglas and Stemerding 2012, Douglas and Stemerding 2013a and b). The *European Policy Workshop* was held in Brussels in March of 2011 with 20 participants that included policymakers and analysts, regulators, members of ethics committees, academics in the social science and philosophy of SynBio, patient organizations, SynBio researchers, and members of the SYBHEL project. The *Global Health Workshop* was held in The Hague in February of 2012, and included 25 participants ranging from representatives from international health organizations and non-governmental organizations, research funders, SynBio practitioners and Do-It-Yourself (DIY) biologists, regulators and biosafety experts, as well as academics in the social science and philosophy of SynBio, and members of the SYBHEL project. These workshops were structured around discussion papers we circulated in advance of the workshop meetings. In these preparatory papers we outlined the possible applications in human health resulting from SynBio, and described the principal governance mechanisms that might be deployed in dealing with the ethical, legal, social and philosophical issues emerging from those applications. In the following discussion of our SYBHEL experiences the initial focus will be on the *European Policy Workshop*. However, in the presentation of our findings and recommendations resulting from the SYBHEL project, we will also include findings from the *Global Health Workshop* and from other work packages in the project.

In preparing for the *European Policy Workshop*, we saw it as our first task to examine a series of reports and articles that identified a variety of potentially promising applications of SynBio in the field of biomedicine and health (NEST High-Level Expert Group 2005; European Group on Ethics of Science and Technology 2009; OECD and The Royal Society 2010; Khalil and Collins 2010; Presidential Commission for the Study of Bioethical Issues 2010; European Academies Science Advisory Council 2010). Based on these documents, prospective future applications could be clustered into a few main areas of application: new pharmaceutical production processes, new kinds of therapeutic and diagnostic products, new forms of targeted delivery, and new options for the development of pharmaceutical products (see Table 1).

**Table 1:** **Potential health applications of synthetic biology**

**Types of application**	**Example**
New pharmaceutical production processes	Synthesis of natural bioactive compounds (Neumann and Neumann-Staublitz 2010), such as artemisinin (Chang and Keasling 2006; Kizer *et al.* 2008; Newman *et al.* 2006; Ro *et al.* 2006)
New kinds of therapeutic products	Engineered viruses and bacteria (Lu and Collins 2009; Forbes 2010)
New kinds of diagnostic products	Biosensors or improved immunoassay (Burbelo *et al*., 2010)
New forms of targeted delivery	Bacteria engineered to invade cancer cells (Anderson et al. 2006)
Development of pharmaceutical products	Synthetically attenuated virus for new vaccines (Coleman *et al.* 2008)

With a general overview of the potentially relevant health applications of SynBio in place we moved to identify the specific ethical and legal issues that might be raised by these applications. These issues were drawn from our own review of the existing policy work in this area (Balmer and Martin 2008; European Group on Ethics of Science and Technology 2009; Royal Academy of Engineering 2009; Rodemeyer 2009; DG 2010; European Academies Science Advisory Council 2010; European Technology Assessment Group and The Rathenau Instituut 2010; Lowerie and Tait 2010; Presidential Commission for the Study of Bioethical Issues 2010; Zhang *et al*. 2011), and were based on the workshop reports of our project partners relating to different philosophical and public understandings of ‘life’ (Deplazes-Zemp 2010 and Deplazes-Zemp 2011), ways in which future advances in SynBio might influence or alter concepts of health, disease, therapy and suffering (Calladine et al. 2010; Szebik 2010; Bradshaw-Martin 2012), and the extent to which current (biotechnology) patent regimes will be constraining or enabling to future SynBio innovation for human health (de Miguel Beriain 2011 and de Miguel Beriain 2012).

To assist us in asking questions about the relationship between SynBio’s prospective health applications and its ELSI issues – as identified from the above sources – we constructed the below Table 2. There the rows refer to the types of SynBio applications identified in Table 1, and the columns reference the general ELSI issues identified in the existing policy work on SynBio. It was not our intention to limit consideration to the kind of ELSI issues that we identified from the existing policy work on SynBio and from our project partners – hence the inclusion of the ‘other?’ category – nor was it our suggestion that one issue applies to only one kind of application. Instead of acting as a rigid analytical framework, these tables were presented as heuristic devices to promote reflection on the relationship between future applications, issues, and their implications for governance. For the use of tables in this regard we took inspiration from the ‘5P governance’ heuristic devised by Alexander Kelle, who identified in this way potential intervention points for biosecurity policy making in SynBio (Kelle 2009: 88).

**Table 2:** **Some ethical, legal, and social issues of synthetic biology health applications**

**Types of SynBio applications**	**ETHICAL, LEGAL, SOCIAL ISSUES (ELSI) OF SYNBIO HEALTH APPLICATIONS**				
	Bio-safety and efficacy of SynBio health products	Dual-use & bio-security issues	Patents and intellectual property	Fundamental ethical questions about engineering (human) life	Other?
New pharma production processes					
New kinds of therapeutic products					
New kinds of diagnostic products					
New forms of targeted delivery					
Development of pharma products					

On the basis of the prospective health applications for SynBio -together with some of the potential ethical, legal, and social issues that they might raise- in the *European Policy Workshop* we hoped to explore various governance approaches as mechanisms that should enable the balancing of possible SynBio benefits vis-à-vis its possible risks and implications. Accordingly, we sought to map these issues on the existing framework of EU policy making, and thereby identify any possible gaps between existing EU governance and SynBio ELSI issues. There are a number of different governance mechanisms available within the European context to address some of the ethical, legal and social issues pertaining to SynBio’s health applications that range from *hard laws* (i.e. directives and regulations) to *soft laws* (i.e. codes of conduct, ethical review, and public engagement), which have been listed in the columns of Table 3. We offered this table as a heuristic aide in our thinking on the ELSI issues listed in the row of Table 3, which map on to those in Table 2.

**Table 3:** **The governance landscape for synthetic biology health application**

**ELSI ISSUE**	**TOOLS FOR SYNBIO GOVERNANCE**				
	Regulations & directives	Research ethics evaluation & funding	Norms, values & professional codes of conduct	Public debate & engage-ment	Other?
Bio-safety and efficacy of SynBio health products					
Dual-Use & Bio-Security Issues					
Patents and intellectual property					
Fundamental ethical questions about engineering (human) life					

The over-arching question guiding the workshop discussions on European governance was: How well do these forms of governance match-up to these ELSI issues? More specifically the following questions were being addressed during the workshop:

- What are the most prominent developments of SynBio likely to make an impact in the area of health in the nearest possible future?
- What are some of the social, ethical, and legal considerations resulting from these SynBio applications in health?
- What tools in the framework of European regulation, governance, and public debate are available to deal with these social, ethical, and legal considerations?
- And following on from that, how can we address these social, ethical, and legal considerations in the European governance framework, and what are the likely outcomes of these social, ethical, and legal challenges for EU health policy?

## Experiences from the European policy workshop: limitations of the ELSI approach

In spite of our preparatory work and use of heuristic devices, our approach and experiences in ‘doing ELSI governance’ did not meet our expectations. Largely this was due to the fact that the future developments that we took as a starting point (in Table 1) relate to potential health applications that are highly uncertain and are likely to appear in society and on the market only in a future time span of decades. As an emerging platform technology SynBio *may* enable a variety of future health applications, but we simply do not yet know where exactly it will lead us, if indeed the promissory visions of its proponents will come to fruition. Thus, during the *European Policy Workshop*, it remained a difficult and open question how to connect (in Table 2) in meaningful ways these highly uncertain and speculative future visions for SynBio to the variety of ELSI issues^a^. Arguably, these general ELSI issues are not particular to SynBio, but refer to broader developments and future visions we are already familiar with from other fields of (bio) technology. In this situation of uncertainty and indeterminacy, we were never really in the position to systematically gauge (in Table 3) the appropriateness of available governance tools for dealing with the future implications of SynBio for human health. Our task of providing meaningful direction for policymakers consequently seemed challenging and severely limited.

Our *European Policy Workshop* was not only instructive in showing the limitations of this ELSI approach, it also made us think about issues of more immediate concern for policy making in the field of SynBio and human health. Main issues of concern that emerged from the workshop discussions were on the one hand related to governmental challenges of SynBio *regulation,* and on the other hand to challenges of anticipatory governance of SynBio *innovation.* In regard to policies of regulation there were discussions about new challenges and needs for supplementary or advanced risk assessment in SynBio, but there were also concerns voiced about the ways in which current regimes of regulation may stifle potentially promising prospects for innovation. In other words, regulation was discussed in the workshop not with respect to prospective future impacts of SynBio, but as part of an ongoing *co-evolution* of science, technology and society. Another important topic emphasized by workshop participants was the need for more inclusive forms of anticipatory governance, involving a variety of actors from both science and society in SynBio innovation. From this perspective the question becomes how to *modulate* ongoing processes of co-evolution by identifying strategic and meaningful choices in the field (Rip 2009). As we learned from our *Global Health Workshop*, the future of SynBio should not only be considered as a series of potential (health) applications, but also as a series of platform technologies which may enable different trajectories of research and innovation (Douglas and Stemerding 2013b).

Although it was our initial intention in the SYBHEL project to discuss the ‘downstream’ implications of future developments in SynBio, the actual workshop discussions made us particularly aware of developments and challenges emerging more ‘upstream’ in the field. As policy analysts we were facing a new and emerging science which is still in an early stage of research and development, and one that is characterized by future uncertainties. Thus, we appropriately decided to steer away in our analysis and policy recommendations from future consequences as our main concern, and instead moved towards more immediate issues relating to current practices and experiences in the field of SynBio and (global) health. In the following we will discuss in more detail our main findings and recommendations based on observations and lessons from both our workshops and from the other SYBHEL work-packages. With these recommendations we want to contribute to a European policy agenda which combines adaptive forms of government regulation (‘hard law’) with forms of anticipatory governance (‘soft law’) in ways that encourage the commitment of both science and society to *responsible research and innovation* in SynBio, acknowledging potential risks without prematurely cutting off developments with potentially significant future (health) benefits.

## Findings and recommendations concerning governmental SynBio regulation

Irrespective of the limitations of our ELSI approach and our initial workshop agenda, the events that we organized did produce inspiring discussions, presentations, and papers. In doing so the workshops helped to open-up SynBio to a wider range of actors and concerns thereby assisting us to translate these concerns into policy recommendations. An important theme arising from the interactions in the *European Policy Workshop* related to the role and limitations of current European GMO safety legislation and medicinal product regulations as they pertain to future health related developments in the field of SynBio. This theme had been previously highlighted by the European Group on Ethics of Science and Technology (EGE) in their official opinion on SynBio (European Group on Ethics of Science and Technology 2009), and we were lucky enough to have three representatives from the EGE in the European Policy workshop. In their report the EGE recommended that the European Commission “should review the legislation applicable to synthetic biology and assess its relevance to address the issues raised by synthetic biology” (2009: 53). In our *European Policy Workshop* several issues were identified as being of special concern in the context of SynBio and human health, from which we derived a number of recommendations that are summarized in Table 4 and will be described in more detail below.

**Table 4:** **Findings and recommendations concerning governmental SynBio regulation**

**Area of regulation**	**Recommendation**
GMO safety regulation	There is a need for dedicated investments in the development of risk assessment methods that match SynBio developments involving the construction of cells with multiple new traits and the introduction of engineered cells or viruses in the human body.
	There is a need to find a new and careful balance within the current system of GMO regulation so that it works to, on the one hand, facilitate innovation and, on the other hand, to prioritize new emerging issues of risk assessment.
Regulation of medicinal products and devices	There is a need to reconsider the current distinction between pharmaceuticals and devices in the process of medical product approval in Europe.
	There is a need to consider a more transparent process of ethical review, in which the results of research ethics evaluations are not only fed back to individual projects (as is the current practice within DG Research and Innovation), but to wider communities of researchers, patients, ethicists and social scientists.

### GMO safety regulation

As the EGE pointed out in its opinion, the aim of SynBio is to produce “organisms with multiple traits from multiple organisms, and therefore it may be difficult to predict their properties” (2009: 49). The problem might even be more complicated as SynBio develops not only engineered bacteria or viruses, but also engineered cells for introduction into humans. As one of the EGE representatives participating in the *European Policy Workshop* noted: the current GM risk assessment paradigm operates on the assumption that small genetic modifications will *not* significantly affect the characteristics of the host organism. However, given the level of complexity in cell engineering and signalling between cells, small changes could have massive ramifications in the human body. As a result serious questions were raised as to whether the current GM risk assessment paradigm is suitable for application in the arena of SynBio and human health. Participants in the European Policy workshop all agreed that there is *a need for dedicated investments in the development of risk assessment methods that match SynBio developments involving the construction of cells with multiple new traits and the introduction of engineered cells or viruses in the human body.*

To be sure new risk management approaches should not be considered in isolation, and discussions took place in other SYBHEL workshops about the need to include regulatory safety concerns in practices of design (Bedau 2011 and Bedau 2012; Deplazes-Zemp 2011). For example, governmental regulations could be used to require the inclusion of a genetic construct preventing uncontrolled reproduction of any engineered organism that could be released into the environment – purposely or otherwise. Prohibiting the approval of any organism that could multiply uncontrollably could work to encourage the kinds of modelling and a design solution proposed by Bedau and others, and represents one way of taking advantage of *regulatory and technological co-production* that is common in the area of health and medicine (Faulkner 2009).

The need for more advanced forms of risk assessment and safety oriented design approaches should however be matched to another concern expressed in the SYBHEL discussions, which relates to the impact on innovation of the current system of GMO regulation. European GMO regulation has developed into a system which is very demanding and costly for innovators in the field, to such an extent that (especially small) innovators may lose interest to pursue potentially promising product developments based on genetic engineering. Maintaining the current system of GMO regulation may thus discourage future SynBio innovation, nipping in the bud promising (health) applications with potential benefits for society, or pushing SynBio research out of the area of human health and into other areas such with lower regulatory hurdles (e.g. cosmetics). From this point of view, European policymaking is at the crossroads for reconsidering the architecture of the current regulatory system. In other words, our workshop participants concluded that there is *a need to find a new and careful balance within the current system of GMO regulation so that it works to, on the one hand, facilitate innovation and, on the other hand, prioritize new emerging issues of risk assessment*.

### Regulation of medicinal products and devices

During our *European Policy Workshop* a representative of the European Medicines Agency (EMA) highlighted the shift that is taking place to biologically-derived medicinal product development, that is, to products based on compounds and cells derived from the human body instead of the conventional chemically-derived compounds that have come to characterize the pharmaceutical industry’s product line. These biologically-derived products create new challenges for the assessment and market approval of medicinal products, which is why particular categories of these products (e.g. gene therapy, cell therapy, and tissue engineering) have been brought into Europe under a special regulatory regime for advanced therapy medicinal products (ATMPs). Currently biologically-derived products only account for 6% of all medicinal drugs on the market in Europe; however, such products may play an increasingly large role as they account for a much larger share of 40% of products in Phase III trials, and health related product innovation in SynBio may further add to this trend (Salmikangas 2011).

A particularly challenging issue in this context is the way in which future (synthetic) biologically-derived medicinal products may increasingly combine different – pharmacological as well as mechanical – modes of action, in which case there would be more and more blurring of the boundaries between distinct regulatory regimes established in Europe for drugs on the one hand, and medical devices on the other. To anticipate future developments in the field of (synthetic) biologically derived products and to strengthen the institutional preparedness of the EMA and other stakeholders in assessing these developments, the workshop found *a clear need to reconsider the current distinction between pharmaceuticals and devices in the process of medical product approval in Europe.*

Other issues raised in the SYBHEL workshop discussions concerned processes of translation for (future) SynBio-based experimental treatments as they move from the laboratory to the clinic. Having patient access to experimental therapies that are actively moving through the translational stages required for formalizing treatments may become a potential source of contention because of the interplay between commercial, individual, public and scientific interests in developing SynBio health applications. Moreover, clinical research needed to develop SynBio health technologies might entail a degree of risk beyond that usually associated with other forms of experimental treatment. Together these issues suggest a need to reconsider the ethical paradigms that apply to clinical research and experimental treatment (Chan 2012). Especially important in this respect is a concern that was expressed in the *European Policy Workshop* about the lack of transparency of the current system of ethical review of individual research projects on the EU level. Thus, in order to support broader ethical reflection on the challenges arising from the development of SynBio therapies and other emerging health technologies, there is *a need to consider a more transparent process of ethical review, in which the results of research ethics evaluations are not only fed back to individual projects (as is the current practice within DG Research and Innovation), but to wider communities of researchers, patients, ethicists and social scientists.*

## Findings and recommendations concerning anticipatory governance of SynBio innovation

Other important reflections arising from the interactions in the *European Policy Workshop* pertained to the nature of SynBio as a science. Due to the fact that SynBio is in an early research stage of development it therefore lacks clearly defined (health) products to which established regulations might apply. Indeed, in several respects, the field is still an ill-defined moving target. A special feature of SynBio is ‘cross-borderness’ (Zhang *et al.*^2011^), or its combination of approaches from a variety of disciplines – biology, engineering, physics, computer science, and chemistry. The field also involves communities of young students attracted by the International Genetically Engineered Machines (iGEM) competition in SynBio, as well as amateur Do-It-Yourself (DIY)Bio or ‘garage’ SynBio communities. Cross-borderness implies new levels of complexity and unfamiliarity, not only in terms of the phenomena studied and engineered in the SynBio field, but also in terms of various disciplinary and cultural traditions crossing each other, with their own norms, values, beliefs, and conventions with regards to risk. In the face of future uncertainty and cross-borderness, we not only need adaptive forms of government regulation, but also a flexible and evolving ‘art of governance’ involving the diversity of communities and stakeholders that are shaping the field (Zhang *et al*. 2011).

Even more important is an *anticipatory* art of governance which takes into account broader discourses of concern, involving deeply rooted convictions and values in society, which may be more decisive for the future social acceptance of SynBio than the more narrowly defined discourses of risk and regulation. In this context, one of the workshop presenters discussed experiences in the UK with a public SynBio dialogue, showing that participants were not only concerned with risks and benefits of potential future applications, but also with the motivations of actors and stakeholders shaping the field, that is, the individual and societal goals and interests that drive research (Bhattachary et al. 2010). Another aspect that was emphasized in the workshop discussions is the need for a shift in focus in these broader discourses of concern from ‘hard’ impacts relating to tangible technological risks for human health and the environment, to ‘soft’ impacts relating to the complex and often unintended ways in which technologies may transform individual behaviours, public attitudes and values, social relationships, and economic balances of power (Swierstra and te Molder 2012).

As was also pointed out in the *European Policy Workshop*, anticipatory governance implies the creation of multiple opportunities for interaction between science and society as a form of ‘extended interdisciplinarity’ (Calvert 2011). This notion implies, on the one hand, engaging communities of researchers with the social and ethical ramifications of their work. On the other hand, it means involving a broad range of stakeholders and publics with issues that relate to current practices and strategies of SynBio research and innovation. In doing so, this interaction process actively distributes the responsibility for the safe and fair development of SynBio practices and products; rather than having such tasks solely allocated to researchers or ethicists (Calvert 2011). Extended interdisciplinary is promoted by the European Commission in terms of ‘responsible research and innovation’, with the aim to integrate and align societal needs and demands more upstream in the technological development process (Von Schomberg 2012 and Von Schomberg 2013; Owen *et al*. 2012). The recent European call for Mobilisation and Mutual Learning Action Plans is a very interesting and significant initiative in this respect. During the *European Policy* and *Global Health Workshops* different options have been proposed that might be supported by the European Commission as *experiments in anticipatory governance*, involving SynBio researchers and a variety of stakeholders and publics in mutual learning about the wider societal aims and concerns in regard to SynBio (and human health)^b^. These recommendations concerning experiments in anticipatory governance are summarized in Table 5 and described in more detail below.

**Table 5:** **Findings and recommendations concerning anticipatory governance of SynBio innovation**

**Type of anticipatory governance**	**Recommendation**
SynBio road-mapping for global health	Organize an open process of community engagement of imaginative thinking for researchers, industry, funding agencies, international health and innovation policymaking organisations, NGOs and representatives of end-users in the field of global public health. Goals would be to outline technical priorities, but also integrating them with research agendas and policy options, to ensure that such an infrastructure would be open, accessible and beneficial for all constructive interests in SynBio, thus stimulating pre-competitive cooperation in the field.
Real-time TA	Engage researchers and a wider circle of relevant stakeholders in an interactive process of imaginative deliberation, considering options, needs, conditions and alternatives for future applications envisaged in particular projects.
Scenarios supporting future imagination	Develop ‘techno-moral’ scenarios as a tool to stimulate imagination, reflection and debate in the context of real time TA, education and public engagement activities.
Experiments with different approaches to intellectual property	Seek to support initiatives from the research community and other parties considering alternative IP regimes and reward structures in SynBio and other fields (e.g. the Health Impact Fund).

### SynBio road-mapping as a governance experiment supporting global health goals

Discussions in the *Global Health Workshop* focused on the ways in which SynBio might address important problems and goals of global public health, as well as the conditions needed to address such issues. One proposal at that workshop suggested that a road-mapping exercise could be conducted for SynBio in global health (Johnson 2012), similar to what has been seen in the case of information communication technologies and semi-conductors (Schaller 2004). Such an exercise could be useful for setting short and longer terms goals, identifying requirements needed for the technology to develop, and coordinate concrete plans of action for the next ten to fifteen years. In doing so it might integrate technical priorities with research agendas and policy priorities, and ensure that such an infrastructure would be open, accessible and beneficial for all constructive interests in SynBio.

The road-mapping exercise proposed by Johnson has some distinctive features compared to the recently published SynBio roadmap for the UK (UK Synthetic Biology Roadmap Coordination Group 2012). The idea of a SynBio roadmap for global health would be to stimulate pre-competitive cooperation in the field and, in doing so, not only circumvent some of the challenges relating to the intellectual property protection of biological parts and processes but simultaneously act as a form of global governance (Johnson 2012). Workshop participants noted that for it to have a chance of being successful it would have to be underpinned by principles of transparency, responsibility and accessibility, and as a result be developed in an open process of community engagement, involving in a process of imaginative thinking, researchers, industry, funding agencies, international health and innovation policymaking organisations, NGOs and representatives of end-users in the field of global public health.

While the idea of a roadmap was broadly supported by *Global Health Workshop* participants, it was clearly not accepted naively. Important considerations about the heterogeneity of SynBio were stressed, which would have to be taken into account if such an exercise were to be conducted. Arguably, a number of different approaches exist for SynBio to contribute to global health (Douglas and Stemerding 2013a). One SynBio researcher participating in the workshop presented a targeted approach in which a specific disease with major global health impacts became the aim of a SynBio funding initiative by the Grand Challenges for Global Health Explorations Grant Program of the Bill and Melinda Gates Foundation (Vohra and Blakely 2013). Another and more generic perspective on addressing global health issues was offered by one of our participants who sought the construction of fundamental multi-purpose pathways in microbial cells, which may serve as platforms that can be tailored to many kinds of applications, using advanced tools for computer-aided design of genetic circuits (Carothers 2013).

Obviously a number of different elements would need to be considered in a roadmapping exercise, and a number of questions remained in the workshop: i.e. who would be involved, who would set its milestones, what matrixes – and underlining values – would be used in evaluation of the milestones and of roadmap(s) more generally, what would be the intellectual property arrangements for any resulting products or processes, would there be only one roadmap? The point here is not to set-out what such an exercise would look like in practice, but rather to offer it as a possible way of governing the development and practice of SynBio for human health. Interestingly, the workshop participant from the OECD Working Party on Biotechnology suggested that this organization might play a role in brokering such an activity. The European Commission and especially the recently established ERA-Net in Synthetic Biology could play a role as well.

### Real-time technology assessment as a governance experiment supporting responsible SynBio innovation

Experiments in anticipatory governance might also include real-time technology assessment (TA) initiatives (Guston and Sarewitz 2002), aiming at mutual learning about challenges relating to the future societal embedding and impacts of potential applications of synthetic biology. The focus of this TA approach is on the level of SynBio research projects, like those funded by the Grand Challenges for Global Health Explorations Grant Program or (health related) projects undertaken as part of the annual iGEM competition. A starting point for a real-time TA is the articulation of future ‘application scenarios’ with the aim to engage researchers and a wider circle of relevant stakeholders in an interactive process of imaginative deliberation, considering options, needs, conditions and alternatives for future applications envisaged in particular projects.

The iGEM community constitutes a particular interesting forum of young researchers in which real-time TA experiments can be – and sometimes are – undertaken in the Human Practice component of the competition. In their work teams of young researchers develop and present proof-of-concept proposals for prospective SynBio applications in the areas of health, environment, food, fuels, and beyond. One of the central components upon which proposals are judged is the degree to which it considers ‘Human Practice’, or the kind of societal impact the prospective SynBio application might have on social organization. This element of the iGEM competition urges participants to reflect on how safety, security, ethics, ownership, sharing, and innovation elements of their prospective intervention will be integrated and received in communities outside of SynBio. In doing so the Human Practice component of the competition asks its young researchers: ‘Will the world be a safe place if we make biology easy to engineer? How do the lessons of the past inform the discussion going forward? Think beyond just convincing people that “synthetic biology is good”. Find a new way to help human civilization consider, guide and address the impacts of ongoing advances in biotechnology’ (iGEM 2012). Not only is a special prize given to the ‘Best Human Practice Advance’, but the gold medal can also be secured if a team ‘outlines and details a new approach to an issue of Human Practice’, alongside the other key judging criteria.

Many of the projects presented at iGEM are associated with larger projects involving senior researchers. While this association stands to benefit and facilitate the development of some of the iGEM projects it also simultaneously creates interesting opportunities to more firmly integrate real-time TA activities in the field of SynBio, thus widening practices of responsible research and innovation. In the process researchers that are new to the field are socialized into a culture of responsibility that facilitates their reflection about the broader contexts into which their future research could be based. Having this reflection take place concurrently with research itself is a key element of real-time TA, and should be supported in this and other spheres of SynBio practice as a positive and productive tool for governance.

### Development of scenarios as a governance experiment supporting future imagination

The development of ‘techno-moral’ scenarios as a tool to stimulate imagination, reflection, debate, education, and public engagement has been proposed in the *European Policy Workshop* as another way to support anticipatory governance in a highly uncertain field. While application scenarios focus on the prospects and challenges for innovation and related regulatory concerns, techno-moral scenarios highlight the wider transformative potential of SynBio in society, thus shifting the attention to ‘soft’ impacts (Swierstra *et al*. 2009; Boenink *et al*. 2010). Techno-moral scenarios invite audiences to imagine and appraise ways in which imperfect technologies like SynBio health applications might change our world, our ideas, values and ideals. As thought experiments techno-moral scenarios can be used to create a controlled environment for testing ideas and arguments about the future. They are useful for exploring and clarify the underlying reasons, beliefs, values and concerns informing public debates about new and emerging science and technology (Calladine and Newson 2011).

Scenario studies are increasingly being used in the area of SynBio (Aldrich *et al*. 2008), and have recently been used by British social scientists who were commissioned by the Biotechnology and Biological Sciences Research Council in the UK to provide a set of tools to engage with the public on issues relating to sustainable bioenergy (Dingwall *et al*. 2011) that interestingly contains a scenario relating to SynBio. Further, the European Patent Office also conducted a large-scale scenario study into how the patent system may be transformed in the near future due to a variety of challenges, which included a scenario on cumulative technologies like SynBio (European Patent Office 2011). Each of these approaches to scenarios studies offers their own particular approach, and target different goals (i.e. reflect on possible changes in values, facilitate public engagement, or anticipate institutional transformation). Nevertheless, these approaches all represent experiments that facilitate imaginative future thinking that can be deployed as a mechanism of governance that seeks to facilitate positive technological development rather than those that attempt to contain future risks.

### Governance experiments supporting different approaches to intellectual property

Issues relating to intellectual property play centrally in both the policy documents outlined in our introduction, and in much of the social science research on SynBio that includes a special issue in the journal *BioSocieties* entirely dedicated to the topic. In their guest editorial Pottage and Marris (2012) stress the importance of not seeing this discourse as a ‘simple dichotomy between open source and patenting strategies’, and instead advocate for social science work into the ‘concomitant engineering of IP and life sciences’. If we accept this view, then governance question becomes: how do we capitalize on the intersections of open source and patenting approaches, as well as the co-production between law and biology, so as to steer SynBio in a positive and productive way?

Throughout our two workshops we found that members of the SynBio research community support the development of a technical infrastructure that is open, accessible and beneficial for all constructive interests in the field. However, as pointed out in one of the other work-package workshops of the SYBHEL project (de Miguel Beriain 2011 and de Miguel Beriain 2012), an important and controversial question still remains: to what extent is it possible to create a robust open access system in a field in which it will be very costly to develop marketable innovations? From our *Global Health Workshop* we learned that SynBio researchers who favour free access to knowledge relevant for global health are facing institutional pressures from their university to pursue IP protection. Given the international IP debate – as discussed for example in the Future Scenarios report of the European Patent Office (European Patent Office 2011) – there is a clear need for flexibility and room to manoeuvre when it comes to IP and patenting for cumulative technologies like SynBio.

As a consequence, experiments with different approaches to IP may function as positive and productive form of governance. For instance, the OECD (2011) recently published a report on collaborative mechanisms for IP management in the life sciences in which they suggest a move away from models of ‘own to protect’ to those of ‘own and share’. Indeed, one of the most notable SynBio applications for global health – i.e. synthetic Artemisinin to combat malaria – has been developed on the basis of such a collaborative arrangement (Douglas and Stemerding 2013b). Another experiment related to IP and public health innovation, that was discussed during the *Global Health Workshop*, was the proposal for a Health Impact Fund (Hollis 2013). The Health Impact Fund leaves the protection and reward components of the patent system intact, and ensures remuneration for the innovators of effective public health interventions for ten years; however, the health interventions themselves are made widely available at cost (Hollis 2008; Hollis and Pogge 2008).

## Conclusion: Achieving policy-relevance in the area of SynBio

In this article we have described how we have shifted our focus in ‘doing ELSI’ over the course of the SYBHEL project. We moved from potential future implications of SynBio for human health as our primary governance concern, to issues of government regulation and anticipatory governance more immediately relating to current practices and experiences in the field. In doing so, our final policy recommendations took the forms that more closely resemble Tables 4 and 5, rather than Tables 2 and 3. This shift in focus is not only relevant in addressing policymaking institutions, but also suggests a need to reconsider established ways of understanding the ethical, legal and social implications of new and emerging science and technology. ELSI research is strongly predicated by a concern to protect society against harmful implications of science and technology and thus reproduces a long-standing division of labour in our society between promoters of new technology and critical responders (Garud and Ahlstrom 1997). In this light, our recommendations for experiments in anticipatory governance imply a move in ELSI research towards a less antagonistic stance, contributing in a critical as well as collaborative way to discussions on how – and under what conditions – SynBio might address important problems and goals of human and global public health. The *Global Health Workshop* that we organized through our SYBHEL work was a most inspiring event in this respect because it clearly showed how the aim of responsible research and innovation might be defined by a common pursuit for the ‘right impacts’. In engaging with a variety of actors related to the research, development, and deployment of SynBio health products, and in constructively reflecting on our own practices of doing so, we have sought to bridge the gap between innovation and ELSI and to produce recommendations that reflect challenges relevant to current practices (yet not limited to the research phases), which are at the same time relevant to – and actionable for – policymakers.

## Endnotes

^a^This was the case despite specific examples of how the way SynBio raised ELSI issue: e.g. horizontal gene transfer from accidental release of engineered bacteria or viruses is a specific bio-safety concern of SynBio health products, synthetically attenuated virus engineering (SAVE) presents real dual-use and bio-security issues, overly narrow or broad patents on essential SynBio building block might stifles health innovation are tangible intellectual property problems, and human enhancement & life extension from SynBio health products pose fundamental ethical questions about engineering -human- life.

^b^Several of the experiments that have been suggested in our workshop discussions have been included in the programme of activities that will be carried out in the next four years by participants of SYNENERGENE, a European FP7 Mobilisation and Mutual Learning Action Plan in the field of synthetic biologie: http://www.synenergene.eu/.
